# Effect of Polyvinyl Siloxane Viscosity on Accuracy of Dental Implant Impressions

**Published:** 2017-01

**Authors:** Ahmad Ghahremanloo, Mahdieh Seifi, Jalil Ghanbarzade, Seyyed Mohammad Abrisham, Rashid Abdolah Javan

**Affiliations:** 1 Associate Professor of Prosthodontics Department, Mashhad Dental School, Mashhad University of Medical Sciences, Mashhad, Iran; 2 Assistant Professor of Prosthodontics Department, Mashhad Dental School, Mashhad University of Medical Sciences, Mashhad, Iran; 3 Assistant Professor of Prosthodontics Department, Yazd Dental School, Shahid Sadoughi University of Medical Sciences, Yazd, Iran; 4 Statistical Advisor, Mashhad Dental School, Mashhad University of Medical Sciences, Mashhad, Iran

**Keywords:** Dental Materials, Dental Implants, Dental Impression Technique, Viscosity, Vinyl Polysiloxane, Dimensional Measurement Accuracy

## Abstract

**Objectives:**

The aim of this study was to compare the accuracy of dental implant impressions obtained by a combination of different impression techniques and viscosities of polyvinyl siloxane (PVS).

**Materials and Methods:**

Four parallel fixtures were placed between mental foramina in a master model of lower dental arch. Three different viscosities (putty/light body, medium body/light body, and monophase: heavy body) and direct and indirect techniques (six groups) were used, and seven impressions were obtained from each group (n=42). To measure the accuracy of impressions, drift, horizontal, and vertical angles of the implants, as well as the hex rotation of the implants in casts were evaluated using a digitizer device (1μm accuracy), in comparison with master arch. Data were analyzed using five-factor two-way ANOVA and Tukey’s post-hoc test.

**Results:**

The accuracy of impressions was assessed and the results showed that direct technique was not significantly different from indirect technique (P>0.05). Also, there were no significant differences between the mentioned viscosities except for the horizontal angle (P=0.006).

**Conclusions:**

Viscosity of impression materials is of high significance for the accuracy of dental impressions.

## INTRODUCTION

Dental implants are increasingly used in clinical practice. They have been of great help in alleviating problems experienced by partially or fully edentulous patients. Today, patients have increasing demands for more appropriate functional and esthetic prostheses [1]. Accuracy is one of the most important factors for the success of implant-supported prosthesis. Prosthesis misfit may cause mechanical problems such as screw loosening, screw fracture and porcelain fracture. Therefore, in order to achieve long-term success, impression accuracy is essential [2–11].

Two basic techniques for dental impression include direct and indirect techniques. Many researchers have evaluated the effects of direct and indirect techniques, splinted and non-splinted techniques, and different impression materials on the accuracy of dental implant impressions [2,7,12–17]. Some researchers believe that direct technique is more accurate than indirect technique [14,15,18]. Ebadian et al, [19] mentioned that using ball-top technique can increase the accuracy of impressions. Another study showed that snap-on technique is as accurate as direct technique [20].

Lee et al, [3] in their systematic review considered the number of implants as an important factor in accuracy of impressions. They concluded that direct technique is more accurate for four or more implants [3]. However, indirect technique might be a better option, particularly in the posterior regions of the mouth, where the impression pin is hardly accessible [21]. Wenz and Hertrampf [22] indicated that a two-step technique with poly vinyl siloxane (PVS) is not suitable for dental impressions. However, there is no consensus regarding the indications for direct and indirect techniques.

To have an accurate impression, not only the impression technique but also the impression material is of great importance. Among different materials, polyether and PVS are considered the most accurate materials for dental impression [2, 23,24].

Sorrentino et al, [16] and also Vojdani et al, [25] concluded that PVS in comparison with polyether has higher accuracy in angulated implants. It has been speculated that a more rigid material would facilitate repositioning of impression coping into the dental impression, given the lower distortion of the material. Walker et al, [21] reported that the viscosity of polyether around impression coping is not of high significance for implant cast accuracy.

To date, no studies have investigated the effects of PVS viscosity on implant cast accuracy. Therefore, the aim of the current study was to compare the accuracy of dental impressions obtained by various combinations of different impression techniques and viscosities of PVS.

## MATERIALS AND METHODS

### Fabrication of the master model:

A master model representing the lower dental arch was fabricated using acrylic resin. At first, four parallel holes were made between mental foramina, using an implant hand-piece with drills, mounted on a dental surveyor. Then, four fixtures 4.1mm wide and 10mm long (IFI 4010PM; DIO, Busan, Korea) were placed in parallel holes (1mm above the alveolar ridge surface) with the help of an impression pin, mounted in a surveyor; afterwards, they were fixed with cold-cure acrylic resin (Acropars, Marlic Company, Karaj, Iran).

**Fig. 1:**
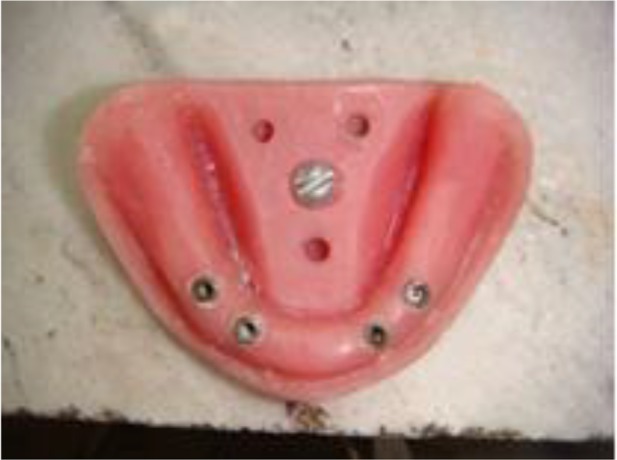
Master arch secured to the table with tripoding

**Fig. 2:**
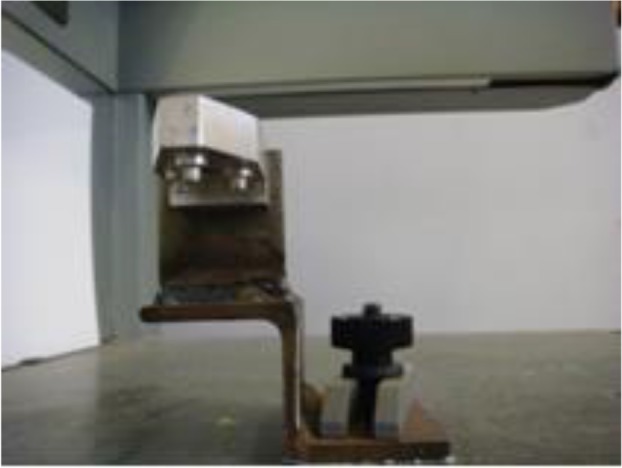
The holder with the base on digitizer machine

Lack of rotation of fixtures was ensured with a torque meter (Digital Force Torque Indicator; Mark-10, NY, USA). Three parallel holes (4 mm wide and 8mm deep) were made in the lingual part of the arch in a triangular shape with a hand-piece mounted on a surveyor to hold the master arch or a cast on a digitizer (measuring machine, S24521; Renishaw Company, New Mills, UK). Afterwards, these three holes (tripod) were scanned using Master Cam X5 software (Master Cam Company, Tolland, USA) to fabricate milled pins, and a holder was used to hold the master arch or cast on the digitizer using a milling machine (CM850; Tabriz Assembling Company, Tabriz, Iran) (Figs. 1 and 2).

### Fabrication of custom trays:

The mentioned master model of dental arch was secured to a table. Then, indirect and direct transfer copings (ITI 48010, IPI 48012; DIO, Busan, Korea) were hand-tightened, and pins were placed in tripod. Two layers of base-plate wax were placed on the master model as a spacer (3mm). Then, two custom trays were made of cold-cure acrylic resin (Acropars; Marlic Company, Karaj, Iran) with 3mm thickness. Two stops on the lingual part of the master arch were considered. In the direct technique, the tray was perforated to access the transfer copings (IPI 48012).

### Impression-taking procedure:

The impression milled pins were placed in the corresponding holes (tripoding), and the impressions were taken with spaced custom trays using two one-step techniques (direct and indirect), three viscosities of PVS (Perfect-f, DIO-Implant; Han DAE Chemical Company, Busan, Korea), and static auto-mixing procedure, performed by an experienced operator.

### The procedure was as follows:

1-Monophase material (heavy-body material, recommended by the manufacturer) was applied on the tray and around the transfer copings.
2-Putty material was applied on the tray and the light-body material was applied around the transfer copings.
3-Medium-body material was applied on the tray and light-body material was applied around the transfer copings.

After the specified time by the manufacturer passed, the impressions were removed and fixture analogues (RMI 48012; DIO, Busan, Korea) were attached and tightened by hand. Then, the casts were poured with vacuum-mixed type 4 stone (Tara; Kheyzaran Company, Isfahan, Iran) according to the manufacturer’s instructions (30g powder/100mL water). The casts were poured immediately after impression taking, were coded, and then measured after 24 hours. In total, for each of the six groups, seven impressions were made (n=42). Sample size was calculated according to the study by Wenz and Hertrampf [22].

### Measurement of accuracy:

At first, the three scanned holes (tripoding) on the master arch were used to fabricate a metal model (holder) to hold the master arch or cast in the same position on the digitizer (Fig. 2).

After the master arches and casts were mounted on the digitizer, different angulations were measured using digitizer probes (Trace Cut version 23; Renishaw Company, New Mills, UK) with the accuracy of 1μm. In the digitizer, analogues were coded 1 to 4, and then, a horizontal line was drawn in the same position as the reference line on the midline of fixture.

**Fig. 3:**
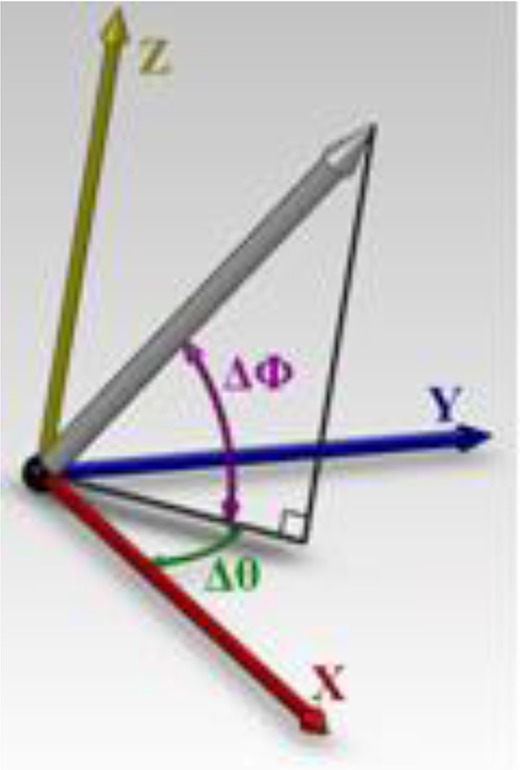
Horizontal (Δθ) and vertical (ΔΦ) angles

Two angles were defined according to the reference line: (1) horizontal angle (Δθ): the angle between the horizontal line and the shadow of the object (abutment) in the horizontal plane; and (2) vertical angle (ΔΦ): the angle between the object (abutment) and the imaginary horizontal plane (Fig. 3).

**Fig. 4:**
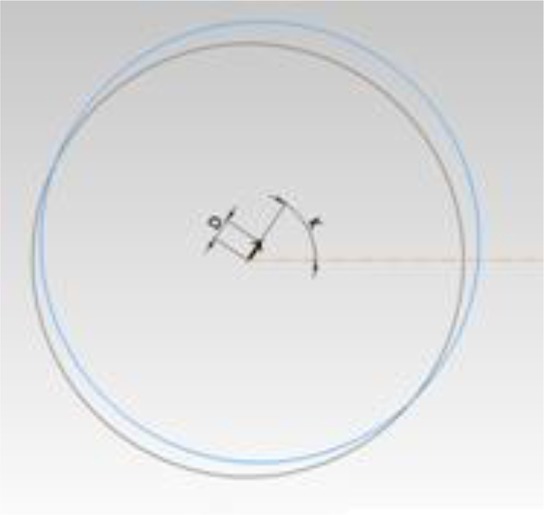
Drift angle in horizontal plane (k)

**Fig. 5:**
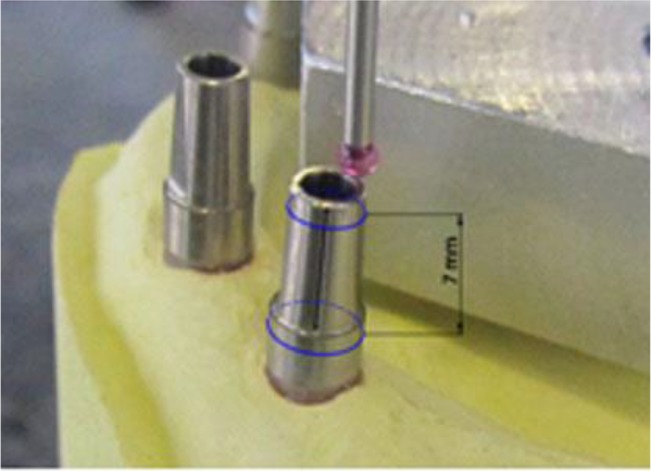
Measurement of abutment direction (vertical and horizontal angles)

**Fig. 6:**
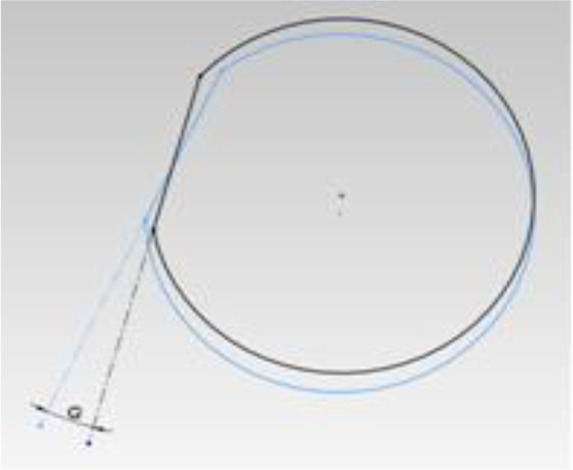
Hex rotation (G)

Overall, four angulations were evaluated (in degrees) including horizontal and vertical angles, drift angle, and hex rotation. For evaluation of the drift angle (k=drift of the analogue center relative to the fixture center in the horizontal plane), the probe touched the edge of the analogue platform and the location of the center was recorded (Fig. 4) (tolerance=0.05°). To measure other angulations (vertical and horizontal angles and hex rotation), the probe touched the abutments, which were tightened with a torque wrench to 35Ncm. To evaluate the direction of the abutments, two margins of the abutments with maximum possible distance (7 mm distance, 1 and 8 mm below the abutment top, respectively) were touched by the probe; the centers of these two circles were connected by a line and this line was called the abutment direction (Fig. 5) (tolerance= 0.02°). To measure the hex rotation (G), the flat surface of the abutment was used (Fig. 6) (tolerance= 0.02°).

The differences of angles between the master arches and casts were calculated by Solid Works 2012 software (Dassault Systems, Concord, MA, USA).

### Statistical analysis:

Seven casts per experimental group sufficed to meet the constraints of α=0.05 and power of 0.80. Data were analyzed by SPSS version 20 (SPSS Inc., IL, USA). For data with normal distribution, Kolmogorov-Smirnov test was applied. Five-factor two-way ANOVA followed by Tukey’s post-hoc test were used for parametric data, and Kruskal-Wallis test followed by pairwise Mann-Whitney test were used for non-parametric data. Also, the mean and standard deviation of four variables were calculated in the six groups using paired t-test.

## RESULTS

Data analysis by Kolmogorov-Smirnov test showed the normal distribution of the data (P>0.05). ANOVA results indicated no significant differences in cast accuracy between direct and indirect techniques and viscosities of impression material, except for the horizontal angle (P=0.006). Also, there were no significant interactions between materials and techniques. Table 1 summarizes the ANOVA results. Table 2 shows the mean and standard deviation of impression accuracy in master arch and casts.

**Table 1:** Results of ANOVA regarding the effects of materials and techniques on the variables

**Source of variation**	**Variables**	**P-value**
**Technique**	Drift angle	0.547
	Vertical angle	0.772
	Horizontal angle	0.401
	Hex rotation	0.405
**Material**	Drift angle	0.100
	Vertical angle	0.159
	Horizontal angle	0.006
	Hex rotation	0.733
**Technique** × **Material**	Drift angle	0.190
	Vertical angle	0.268
	Horizontal angle	0.871
	Hex rotation	0.367

**Table 2:** The mean and standard deviation of angular differences (in degrees) between the master arch and casts

**Variable**	**Technique**	**Groups according to technique**				

		**Group 1**	**Group 2**	**Group 3**
**Drift angle**	Direct	126.26±30.88	68.17±42.41	110.50±46.56
	Indirect	87.16±30.50	87.16±38.02	107.19±55.53
**Vertical angle**	Direct	0.50±0.19	1.07±0.81	0.66±0.29
	Indirect	0.60±0.34	0.77±0.31	1.00±0.79
**Horizontal angle**	Direct	8.66±2.90	16.52±8.25	13.94±4.69
	Indirect	8.12±2.43	13.89±8.07	12.83±2.73
**Hex rotation**	Direct	6.59±6.41	5.03±5.10	9.22±7.36
	Indirect	10.50±6.54	8.39±7.63	6.90±4.47

## DISCUSSION

The first step in fabricating implant-supported prosthesis is taking a dental impression. An accurate impression can help us transfer the correct position of the implants to the laboratory to achieve passive fit of restoration; it can also prevent biological and mechanical complications [3,4]. To achieve passive fit, the summation of distortion equation should come to zero. It includes the following clinical and laboratory procedures: impression taking, master cast fabrication, wax pattern fabrication, framework fabrication, definitive prosthesis fabrication and definitive prosthesis delivery [11].

In this study, different viscosities of PVS were used for taking dental impressions. According to the results, different PVS viscosities have the same accuracy to record drift angle, vertical angle, and hex rotation. However, monophase material was more accurate than a combination of putty and light-body materials in recording the horizontal angle.

As shown in the study by Walker et al, [21] no significant difference was observed in evaluating the horizontal position of the implants. In their study, they used a microscope with the accuracy of 1μ, similar to the current study; although four angulations were evaluated in the present study. The lower accuracy of putty/light-body materials in the horizontal angle could be due to increased content of fillers, which causes more significant rebound effects [26], or the limited space in the custom trays; however, in the study by Wenz and Hertrampf [22] 3mm space was considered acceptable in custom trays.

Generally, there are two impression techniques in implant-supported restorations namely direct and indirect. Each technique has some advantages and disadvantages [9]. In the current study, there was no significant difference between direct and indirect impression techniques, similar to some previous studies [2,6,12]. However, according to some previous studies, direct impression technique is more accurate than the indirect technique [7,15,18,21]. In some studies, the higher accuracy of direct impression technique, (compared to indirect technique) may be due to placing the transfer coping in the correct position. Mostafa et al, [15] stated that permanent distortion during the movement of transfer copings through large undercuts can lead to inaccuracy. Higher distortion in the impression is associated with lower retention of indirect copings in the impression; therefore, there is a high possibility that the coping is placed in a wrong position.

Daoudi et al, [13] in their study indicated that none of the three study groups (dentists, residents, and technicians) could place the transfer coping in the correct position, using the indirect technique. They mentioned that incorrect positioning of transfer coping was the primary reason for errors in the indirect technique. It may be stated that in the indirect technique, distortion of impression material and incorrect positioning of transfer copings may lead to inaccuracy.

Also, analogues are assembled out of the dental impression and seem to be more visible and accurate. However, assembling analogues in the direct technique is more difficult, especially in case of deep analogues. In addition, transfer coping in the impression material may move during transfer pin opening or closing [6,8].

In our study, four variables were measured for the evaluation of dental impressions, unlike several studies which have measured only one variable. In the study by Akca and Cehreli [2], the upper part of hex and implant angulations were measured. In the current study, the hex rotation was evaluated for the first time. Apparently, this factor is quite important for supra-structure passivity, and its absence may cause mechanical and biological problems.

In similar studies [21,22], different intervening factors, e.g., movement of master arch during impression, impression tray, mixing methods, and methods of measurement have been mentioned. In our study, we aimed to minimize these factors. During the removal of the tray off the master arch, imbalanced forces may affect the impression material [15]. Therefore, there might be some distortions in some areas of the material. In our study, in order to minimize this intervening factor, the master arch was secured to a table so that the tray could be removed more easily. Type of the tray is another factor influencing the accuracy of dental impressions. Normally, steel stock trays are more rigid than custom trays; however, uniform thickness of impression material cannot be ensured in all areas in the stock trays, which leads to uneven polymerization shrinkage. Therefore, a 3mm-spaced custom tray with 3mm thickness was used to obtain acceptable rigidity and a uniform thickness in every impression. However, some studies reported no difference between stock and custom trays [2,21].

In our study, a holder was used to hold the master arch or cast on the digitizer surface. By the help of this holder, intervening factors associated with casts such as trimming angulations and height of the cast bases were eliminated. Ebadian et al, [19] considered the center of fixture or analogue as the reference point for calibration of measurements. However, its change can flaw the measurement. In our study, the reference point was on the cast with less changes. Also, auto-mixing method was used for preparing impression materials in our study. This method is the most commonly used method in clinical practice and eliminates the improper base/catalyst ratio during mixing.

Measuring the angulations on abutments would be more accurate as evaluations are done out of the fixtures/analogues. However, there are some dimensional differences in abutments made by a company. To date, no study has evaluated the effect of these differences on clinical procedures. The milled pins used in this study may be dislodged during impression taking. These can also be made by milling machine for more accuracy. It is recommended to carry out more studies with other materials and implant systems on master arches for prepared teeth or partially edentulous arches. Also, the soft tissue should be simulated in future studies. Dimensional differences of an implant part manufactured by a company should be taken into consideration as well.

## CONCLUSION

Considering the limitations of this study, there were no significant differences in the accuracy of dental implant impressions between direct and indirect techniques or different PVS viscosities. However, mono-phase recorded the horizontal angle more accurately than the combination of putty/light-body materials.
